# Diagnostic efficacy of the triglyceride–glucose index in the prediction of contrast-induced nephropathy following percutaneous coronary intervention

**DOI:** 10.3389/fendo.2023.1282675

**Published:** 2023-11-22

**Authors:** Wei−Ting Chang, Chien-Cheng Liu, Yen-Ta Huang, Jheng-Yan Wu, Wen-Wen Tsai, Kuo−Chuan Hung, I−Wen Chen, Ping-Hsun Feng

**Affiliations:** ^1^ School of Medicine and Doctoral Program of Clinical and Experimental Medicine, College of Medicine and Center of Excellence for Metabolic Associated Fatty Liver Disease, National Sun Yat-sen University, Kaohsiung, Taiwan; ^2^ Division of Cardiology, Department of Internal Medicine, Chi-Mei Medical Center, Tainan, Taiwan; ^3^ Department of Biotechnology, Southern Taiwan University of Science and Technology, Tainan, Taiwan; ^4^ Department of Anesthesiology, E-Da Hospital, I-Shou University, Kaohsiung, Taiwan; ^5^ Department of Nursing, College of Medicine, I-Shou University, Kaohsiung, Taiwan; ^6^ School of Medicine, I-Shou University, Kaohsiung, Taiwan; ^7^ Department of Surgery, National Cheng Kung University Hospital, College of Medicine, National Cheng Kung University, Tainan, Taiwan; ^8^ Department of Nutrition, Chi Mei Medical Center, Tainan, Taiwan; ^9^ Department of Neurology, Chi-Mei Medical Center, Tainan, Taiwan; ^10^ Department of Anesthesiology, Chi Mei Medical Center, Tainan, Taiwan; ^11^ School of Medicine, College of Medicine, National Sun Yat-sen University, Kaohsiung, Taiwan; ^12^ Department of Anesthesiology, Chi Mei Medical Center, Liouying, Tainan, Taiwan

**Keywords:** contrast-induced nephropathy, triglyceride-glucose index, meta-analysis, insulin resistance, cardiovascular disease

## Abstract

**Introduction:**

Contrast-induced nephropathy (CIN) is a common complication of percutaneous coronary intervention (PCI). Identifying patients at high CIN risk remains challenging. The triglyceride-glucose (TyG) index may help predict CIN but evidence is limited. We conducted a meta-analysis to evaluate the diagnostic value of TyG index for CIN after PCI.

**Methods:**

A systematic literature search was performed in MEDLINE, Cochrane, and EMBASE until August 2023 (PROSPERO registration: CRD42023452257). Observational studies examining TyG index for predicting CIN risk in PCI patients were included. This diagnostic meta-analysis aimed to evaluate the accuracy of the TyG index in predicting the likelihood of CIN. Secondary outcomes aimed to assess the pooled incidence of CIN and the association between an elevated TyG index and the risk of CIN.

**Results:**

Five studies (Turkey, n=2; China, n=3) with 3518 patients (age range: 57.6 to 68.22 years) were included. The pooled incidence of CIN was 15.3% [95% confidence interval (CI) 11-20.8%]. A high TyG index associated with increased CIN risk (odds ratio: 2.25, 95% CI 1.82-2.77). Pooled sensitivity and specificity were 0.77 (95% CI 0.59-0.88) and 0.55 (95% CI 0.43-0.68) respectively. Analysis of the summary receiver operating characteristic (sROC) curve revealed an area under the curve of 0.69 (95% CI 0.65-0.73). There was a low risk of publication bias (p = 0.81).

**Conclusion:**

The TyG index displayed a noteworthy correlation with the risk of CIN subsequent to PCI. However, its overall diagnostic accuracy was found to be moderate in nature. While promising, the TyG index should not be used in isolation for CIN screening given the heterogeneity between studies. In addition, the findings cannot be considered conclusive given the scarcity of data. Further large-scale studies are warranted to validate TyG cutoffs and determine how to optimally incorporate it into current risk prediction models.

**Systematic Review Registration:**

https://www.crd.york.ac.uk/prospero/display_record.php?ID=CRD42023452257, identifier CRD42023452257.

## 1 Introduction

Contrast-induced nephropathy (CIN), characterized by rapidly declining renal function following the administration of iodinated contrast agents, is the most commonly encountered dilemma during percutaneous coronary intervention (PCI) (1–4). CIN is associated with poor clinical outcomes including long-term deterioration in kidney function and increased mortality risk (5–7). Therefore, identifying patients at high risk of CIN is crucial to optimize prevention strategies and clinical management. Predicting CIN involved assessing risk factors and identifying high-risk patients, which remains challenging (1, 3, 8). To date, although pre-existing kidney disease, diabetes, advanced age, hypertension, and heart failure are reported as risk factors of CIN, whether other index also contribute to the development of CIN is still largely unknown (1, 2, 8).

Research interest has recently focused on exploring whether certain metabolic markers could further refine CIN risk stratification. The triglyceride–glucose (TyG) index, which is determined by fasting triglyceride and glucose levels, has been regarded as a marker representing insulin resistance and metabolic status (9–11). TyG index was calculated as the Ln[fasting triglycerides(mg/dl) × fasting glucose (mg/dL)/2]. Studies have suggested that the TyG index could serve as a predictive marker for cardiovascular risk such as coronary artery disease, heart attack, and stroke (9, 10, 12). In addition, some studies have explored the potential connection between the TyG index and nephropathy, particularly in diabetic nephropathy (13–17). A higher TyG index, indicative of insulin resistance, can contribute to inflammation, oxidative stress, and vascular dysfunction (9, 15, 18). In addition, the TyG index exhibits a positive correlation with a high risk of developing diabetic nephropathy and other renal-related complications (15, 17).

Emerging evidence suggests that the TyG index may predict the risk of CIN (13, 14). For instance, Aktas et al. found that the TyG index was an independent predictor of CIN in 272 patients without diabetes who were undergoing PCI for non-ST elevation myocardial infarction (13). In another study of 350 patients without diabetes who were undergoing PCI for acute coronary syndrome, Gursoy and Baydar observed that the incidence of CIN was significantly higher in those with high TyG indices (≥8.65) than in those with lower indices (<8.65) (14). Importantly, the predictive value of the TyG index for CIN appears to be present even after adjusting for potential confounders such as diabetes status (13, 14). While promising, research on the TyG index and CIN risk remains limited. Only a few studies, mainly from China and Turkey, with relatively small sample sizes, have examined this association (13–17). Moreover, the diagnostic accuracy of the TyG index remains unclear. Further investigation through meta-analyses could help synthesize the current evidence regarding the utility of the TyG index as a predictor of CIN in patients undergoing PCI. Thus, in this diagnostic meta-analysis, we aimed to investigate whether the TyG index could be used to predict the likelihood of CIN in patients receiving PCI.

## 2 Methods

### 2.1 Protocol registration

This report was presented in compliance with the PRISMA guidelines and registered in PROSPERO (CRD42023452257).

### 2.2 Literature search

Potentially relevant articles were identified following searches of electronic databases such as MEDLINE, Cochrane Library, and EMBASE, spanning from their inception to August 3, 2023. The search was conducted using relevant keywords: (“PCI” or “Left Main Disease” or “Coronary Angiograph*” or “coronary artery disease” or “Percutaneous Coronary Intervention” or “Myocardial infarction” or “MI”) and (“Triglyceride–Glucose Index” or “Triglyceride–Glucose Indices” or “TyG” or “insulin resistance”), and (“CI-AKI” or “CA-AKI” or “Contrast-induced acute kidney injury” or “Contrast-Associated Acute Kidney Injury” or “Contrast-Induced Nephropathy” or “Contrast-Associated Nephropathy” or “Acute Renal Insufficiency” or “Acute Kidney Insufficiency” or “Renal Insufficienc*” or “Kidney insufficienc*” or “Acute kidney injury” or “AKI” or “Nephropathy”). Using a comprehensive approach, controlled vocabulary search terms were used alongside keyword searches to enhance the breadth of the literature exploration. Furthermore, the reference lists from the acquired studies were manually checked to find potentially suitable articles. The search was comprehensive and was not limited by country or language of publication. **Supplementary Table 1** provides detailed information of the search strategies.

### 2.3 Inclusion and exclusion criteria

In this meta-analysis, the selection of studies for inclusion adhered to predefined criteria: (1) the studies encompassed patients with coronary disease who had undergone coronary angiography or PCI, (2) the studies employed the TyG index as a predictive measure for CIN risk, and (3) these studies furnished comprehensive data concerning sensitivity, specificity, and count of patients with CIN. Randomized controlled trials or retrospective study designs (e.g., case–control studies) were deemed suitable for this meta-analysis.

Studies were excluded if (1) they were exclusively presented as conference papers, case series, abstracts, or review articles; (2) they concentrating on outcomes other than CIN; and (3) they did not provide full-text versions.

### 2.4 Data retrieval

Two team members extracted information separately from the individual research papers, and any differences were resolved through discussion. To gather any missing details, efforts were made to communicate with the corresponding author of the studies. The extracted data for each study included the country of origin, author’s details, sample size, study setting, age, sex, information on specificity/sensitivity values, details related to the TyG index, and count of patients with CIN.

### 2.5 Outcomes

This diagnostic meta-analysis was conducted to appraise the precision of the TyG index in anticipating the probability of CIN, as per the definitions employed in individual studies. Secondary outcomes sought to appraise the combined incidence of CIN and the relationship between a high TyG index and the risk of CIN.

### 2.6 Quality assessment

The Quality Assessment for Diagnostic Accuracy Studies-2 (QUADAS-2) tool was used to assess the risk of bias (19). Four main domains including patient selection, index test, reference standard, and flow and timing were systematically examined to identify sources of bias. Two authors conducted a subjective review of all eligible studies to establish consistency. By using the QUADAS-2 tool, the authors assigned each domain to one of three categories: ‘low risk,’ ‘some concerns,’ or ‘high risk. Discussions were held to resolve discrepancies.

### 2.7 Statistical analysis

For all statistical analyses, Stata version 15.0 and RevMan version 5.4 were used. The sensitivity and specificity were extracted from each study and pooled. Heterogeneity was assessed using the I^2^ statistic, where an I^2^ >50% signifies significant heterogeneity. Publication bias was evaluated through Deeks’ funnel plot asymmetry test. To summarize the diagnostic accuracy, a hierarchical summary receiver operating characteristic curve was constructed, and the area under the curve (AUC) was calculated. Fagan’s nomogram was utilized to estimate post-test probability based on likelihood ratios (LRs) (20). A positive LR in the range of 2–5 slightly increase the likelihood of disease presence following the test, whereas ratios ranging from 5 to 10 moderately enhance the likelihood; ratios >10 significantly amplify the probability of having the disease (21). The predictive utility of the TyG index was assessed by examining how the post-test probability changed from the pre-test probability. Two-sided tests in all statistical analyses were conducted at a significance level of 0.05.

## 3 Results

### 3.1 Database searching and study characteristics

**Figure 1:**
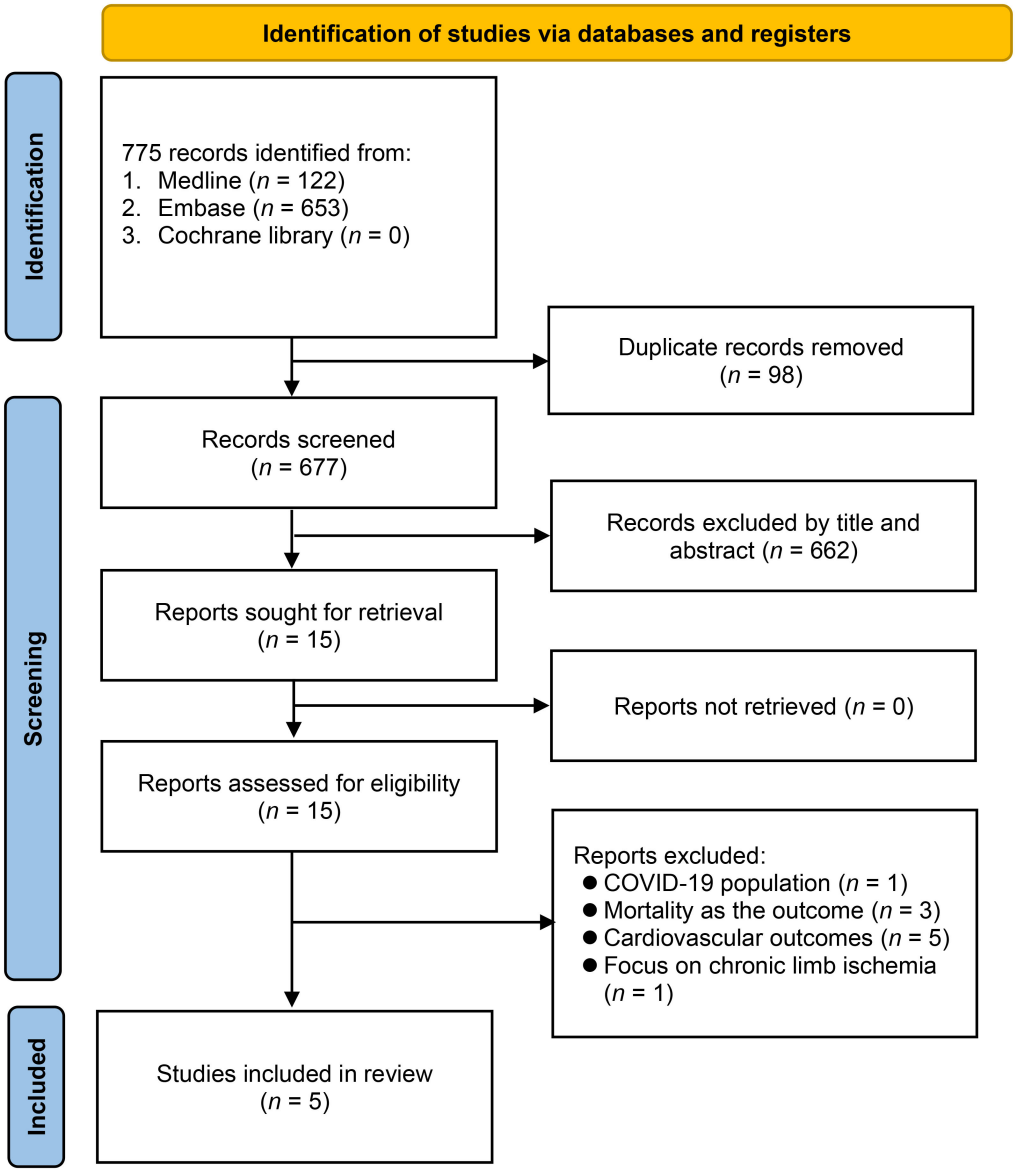
Flow chart for study selection.

Through a search on four electronic databases, 890 records were discovered (**Figure 1**). By eliminating duplicate studies (n = 130) and carrying out initial title and abstract examination (n = 760), 15 articles emerged as potentially aligned with the predefined inclusion criteria. Subsequent full-text reading led to the exclusion of 10 studies. Ultimately, five studies met the predetermined requirements and were included in the meta-analysis (13–17).

The characteristics of **s**tudies involving 3518 patients are summarized in **Table 1**. All studies were conducted in two countries, including Turkey (n = 2) (13, 14) and China (n = 3) (15–17). In these studies, the age range of the patients fell between 57.6 and 68.22 years. The number of participants varied across the studies, ranging from 272 to 1108, with male proportions ranging from 52.3% to 79.4%. Among the five studies, two on patients without diabetes mellitus (DM) (13, 14), while two targeted patients with DM (15, 17). However, one study did not explicitly specify this information (16). Regarding the definition of CIN, three studies adopted the same criteria, defining CIN as either a 25% increase or a 0.5 mg/dL increase in serum creatinine (Scr) levels from baseline within the first 48–72 h (13, 14, 16). In another study, CIN was defined as a 50% increase or a ≥0.3 mg/dL increase in Scr levels from baseline within 1 week (15). However, one study did not specify the criteria used to define CIN (17). The AUC, which reflects the accuracy of the predictive models, ranged from 0.662 to 0.811. The sensitivity varied between 59.3% and 94.9%, while the specificity ranged between 31.7% and 72%. Four studies (13, 14, 16, 17) reported cutoff values to predict CIN occurrence, with the range being 8.69–9.17, while one study did not provide this information (15).

**Table 1:** Characteristics of studies (n = 5).

Parameters	Aktas 2023	Cursoy 2023	Hu 2022	Li 2022	Qin 2021
Age (years)	60.47 ± 12.25	57.6 vs 56.5	65 (56-71)	66.4 ± 10.4	68.22 ± 10.6
Male (%)	79.4%	52.3%	62.4%	62.5%	68.7%
BMI (kg/m^2^) or weight (kg)	27.2 ± 3.3	76 vs. 76	24.5 (23-26.8)	24.9 ± 3.2	25.0 ± 3.7
N	272	350	860	1108	928
Population	Non-DM patients with NSTEMI	Non-DM patients with NSTEMI	DM patients with ACS	Patients with NSTE-ACS	DM patients with MI
CIN %	6.6%	16%	19.9%	15.1%	21.2%
Definition of CIN	A 25% or 0.5 mg/dL increase in Scr from baseline within the first 48-72 hours.	A 25% or 0.5 mg/dL increase in Scr from baseline within the first 48-72 hours.	A 50% or ≥0.3 mg/dL increase in Scr from baseline within one week	A 25% or 0.5 mg/dL increase in Scr from baseline within the first 48-72 hours.	Based on the preoperative and postoperative Scr, as measured within one week
AUC	0.712	0.666	0.811	0.662	0.728
Sensitivity	61%	71.4%	77.9%	59.3%	94.9%
Specificity	72%	55.1%	31.7%	66.1%	51.3%
Cut-off values for TyG index	9.17	8.69	NA	9.043	8.88
Country	Turkey	Turkey	China	China	China

Scr, serum creatinine; CIN, contrast-induced nephropathy; TyG, triglyceride-glucose; BMI, body mass index; AUC, area under curve; DM, diabetes mellitus.

### 3.2 Risk of bias

**Figure 2:**
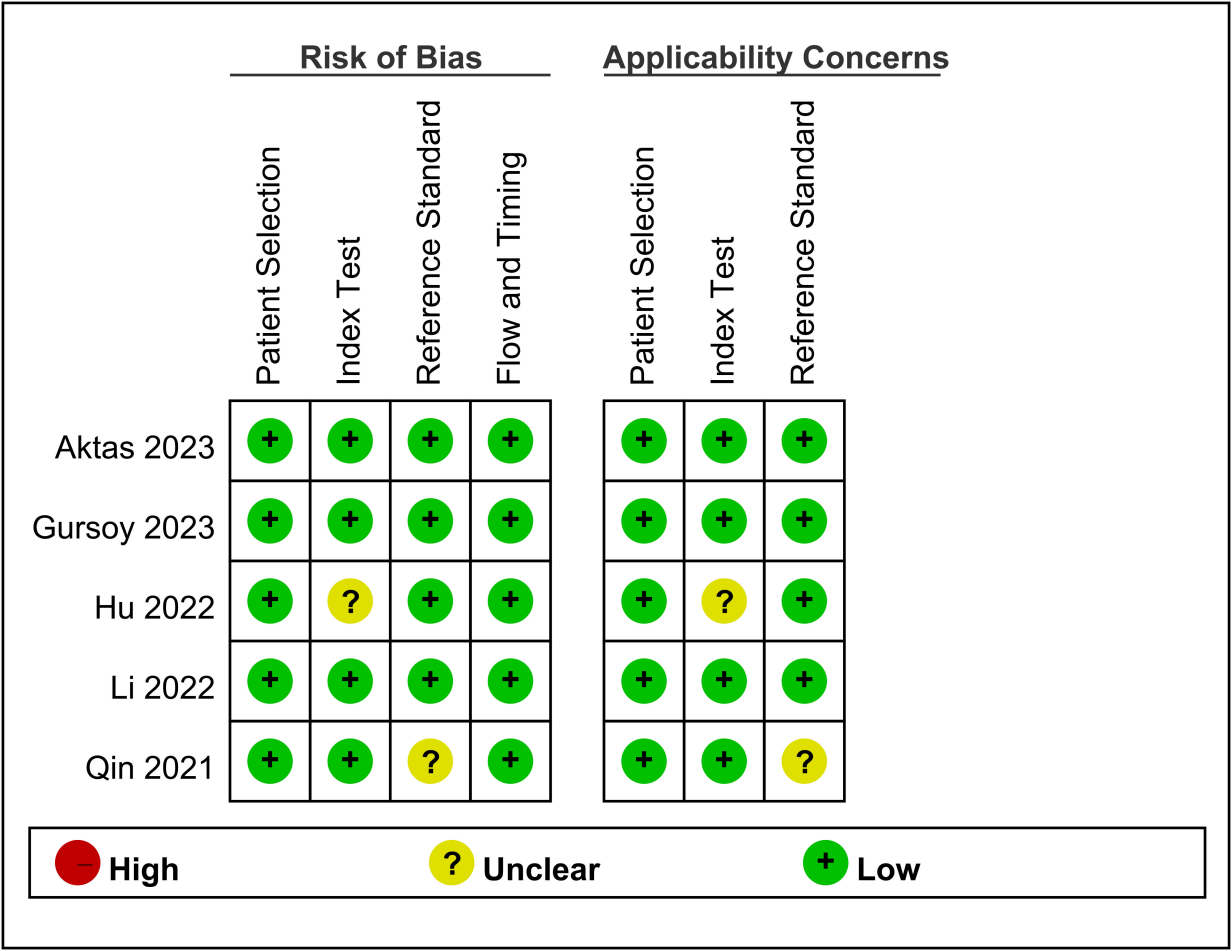
Methodological quality summary of included studies.

The risk of bias and applicability concerns for the five included studies are shown in **Figure 2**. Overall, the majority of studies displayed a low risk of bias across the assessed domains. The primary areas of concern were related to the measurement of the TyG index in one study (15) and the definition of CIN in another (17). Apart from these issues, patient selection, index tests, reference standards, and flow/timing exhibited a low risk of bias in the included studies.

### 3.3 Outcomes

#### 3.3.1 Pooled incidence of CIN

**Figure 3:**
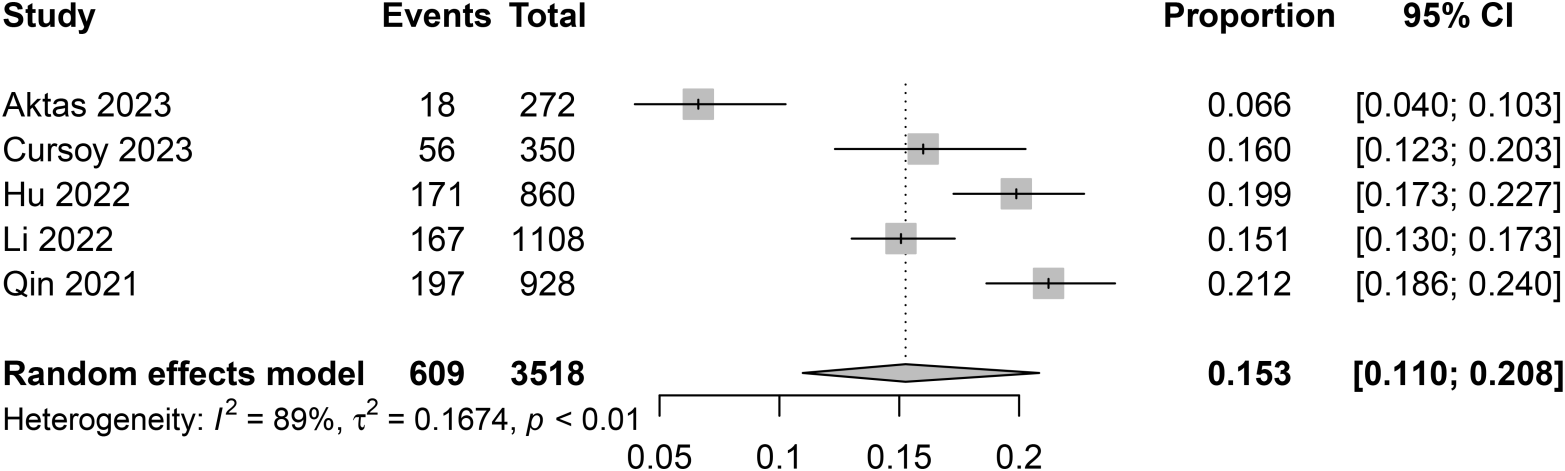
Pooled incidence of contrast-induced nephropathy (CIN).

The incidence of CIN varied among studies, with the lowest being 6.6% in a study from Turkey (13), and the highest reaching 21.2% in a study conducted in China (17). After pooling data from a total of 3518 patients, the combined incidence of CIN was 15.3% (95% confidence interval [CI] 11% to 20.8%) (**Figure 3**).

#### 3.3.2 Association between the TyG index and CIN risk

**Figure 4:**
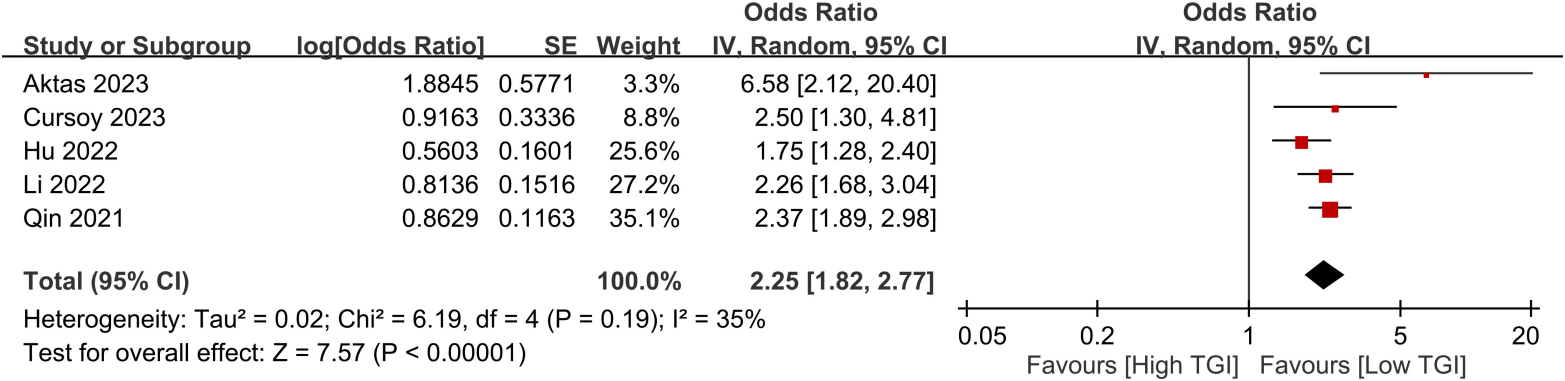
Forest plot showing a positive relationship between a high triglyceride–glucose (TyG) index and the risk of contrast-induced nephropathy.

Five studies consistently demonstrated a positive relationship between the TyG index and CIN risk. Upon calculation, a high TyG index was associated with an increased risk of CIN, with an odds ratio of 2.25 (95% CI 1.82–2.77, p < 0.00001) (**Figure 4**). The level of heterogeneity across these studies was moderate (I^2 =^ 35%).

#### 3.3.3 Diagnostic efficacy of the TyG index for CIN

**Figure 5:**
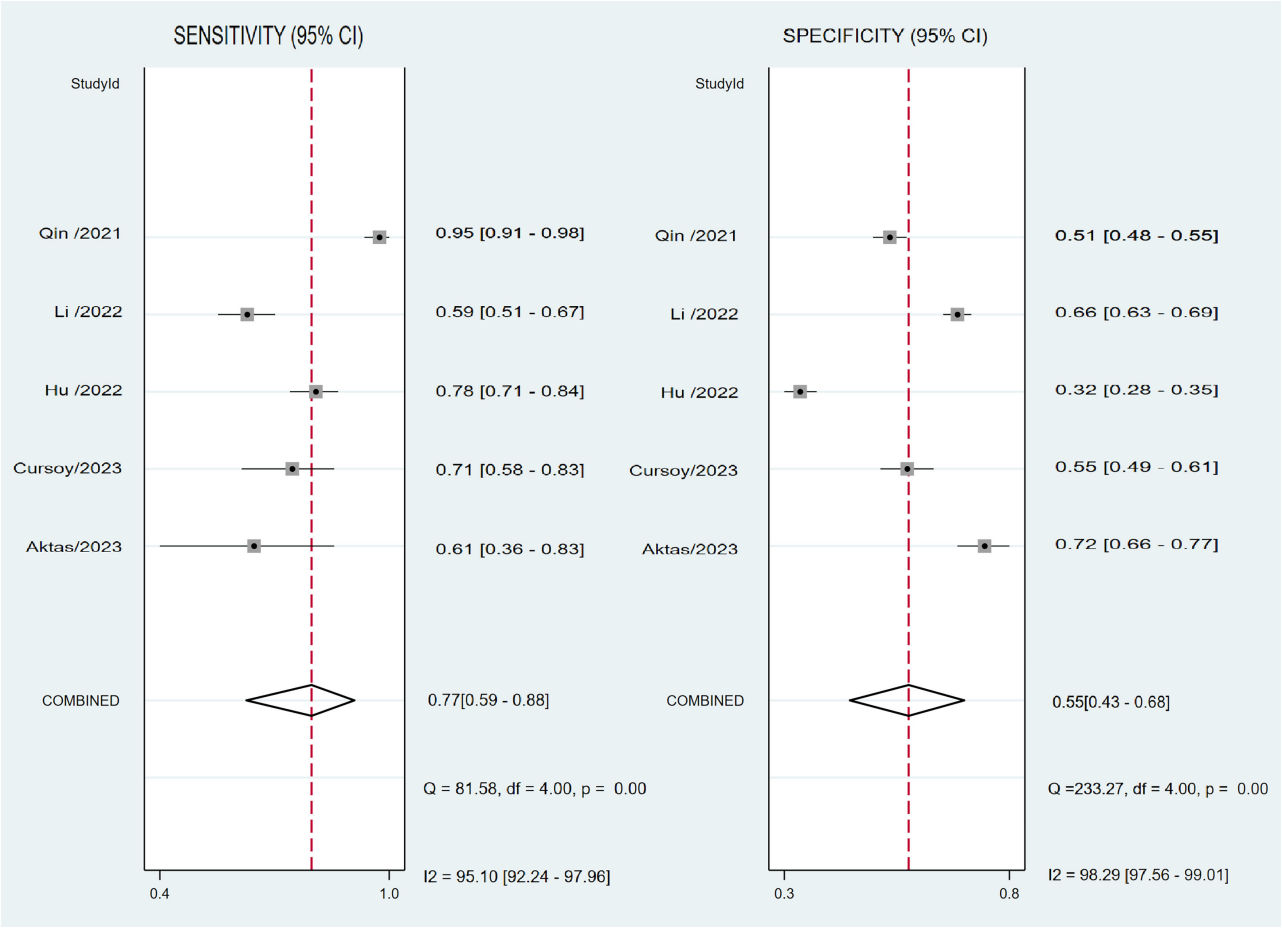
Forest plot depicting the combined sensitivity and specificity of the triglyceride–glucose (TyG) index in predicting contrast-induced nephropathy.

**Figure 6:**
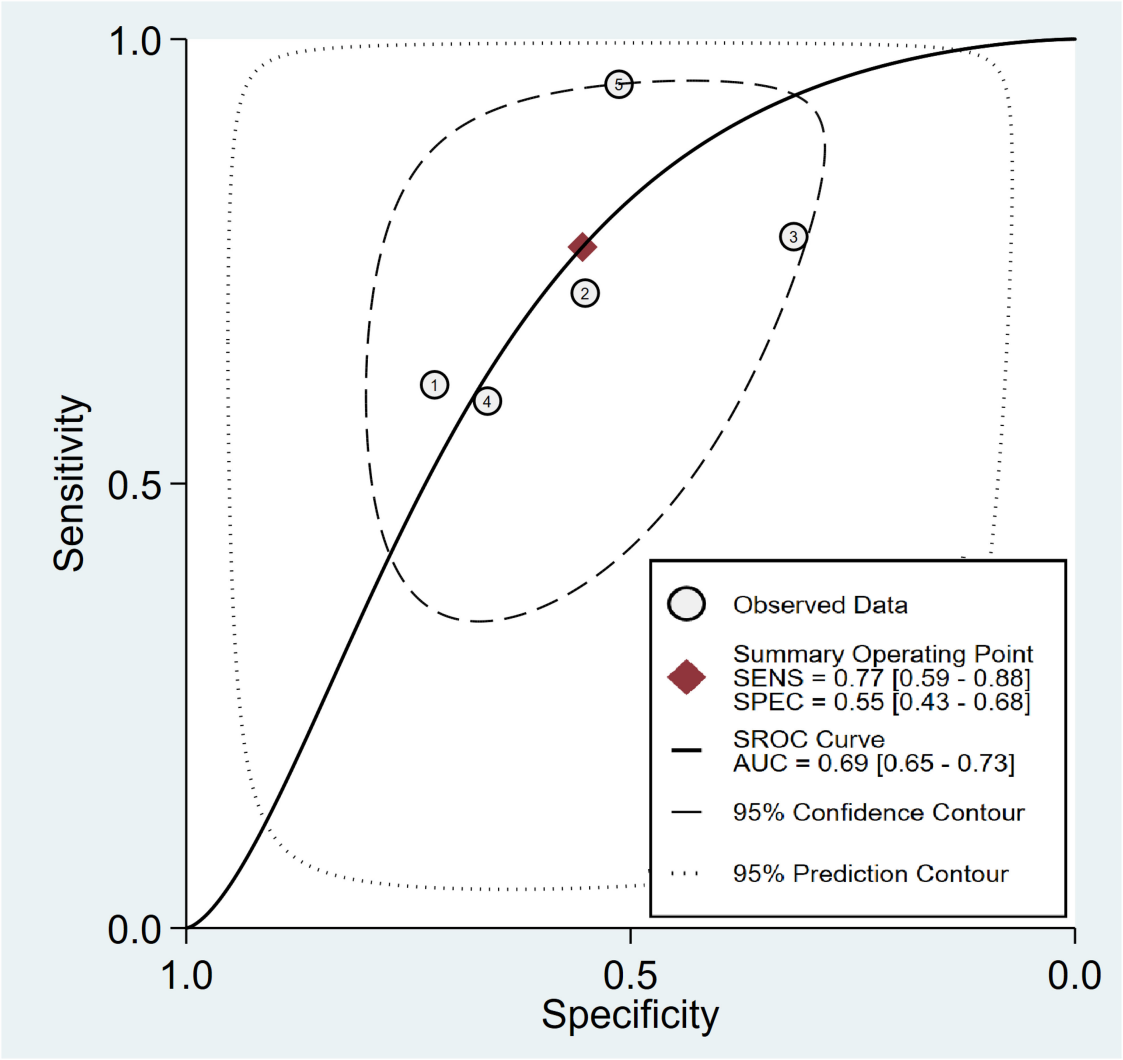
Analysis on summary receiver operating characteristic (sROC) curve demonstrating the predictive effectiveness of the triglyceride–glucose Index concerning contrast-induced nephropathy.

**Figure 7:**
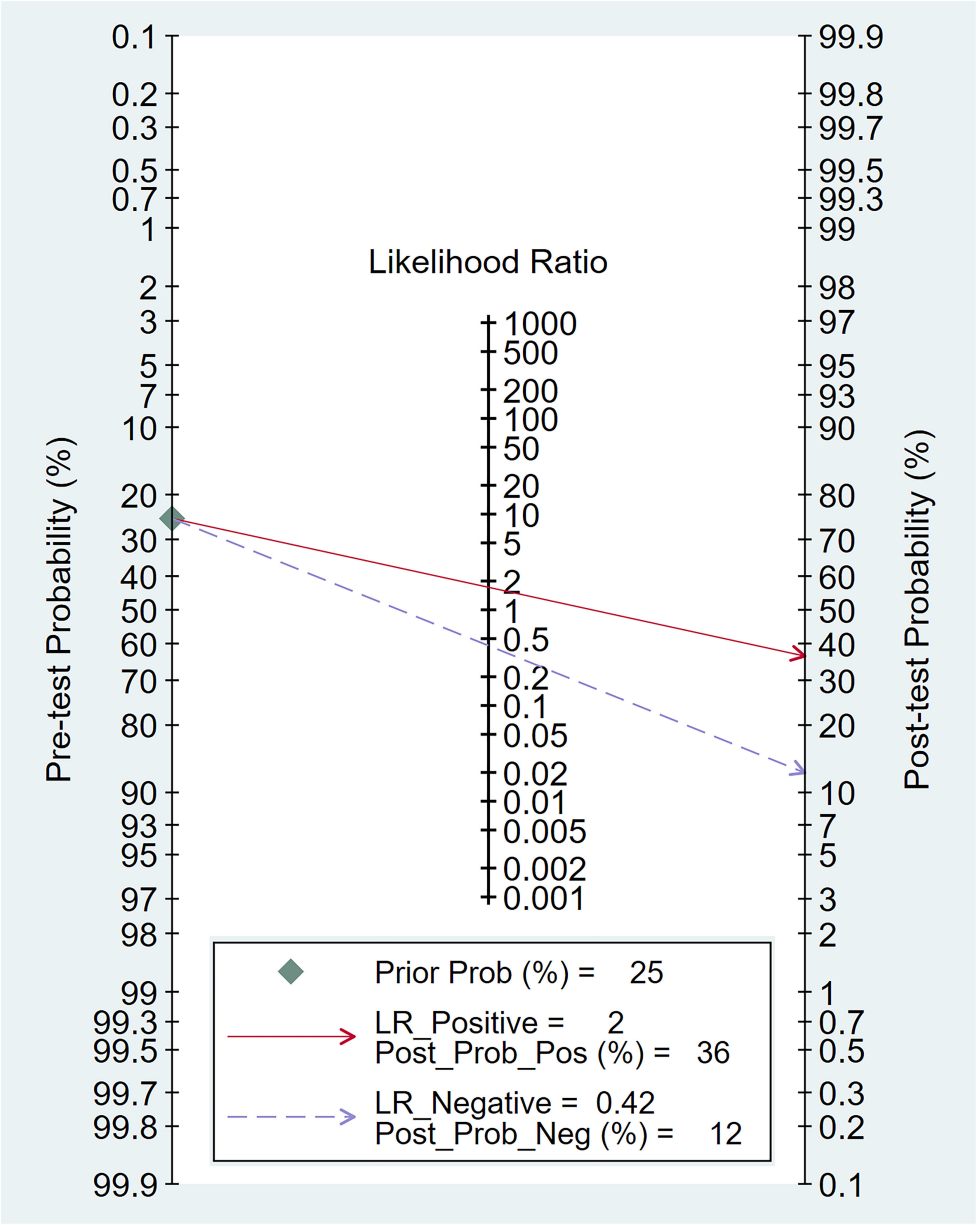
The clinical applicability of the triglyceride–glucose (TyG) index in forecasting the occurrence of contrast-induced nephropathy illustrated through Fagan’s nomogram plot.

**Figure 8:**
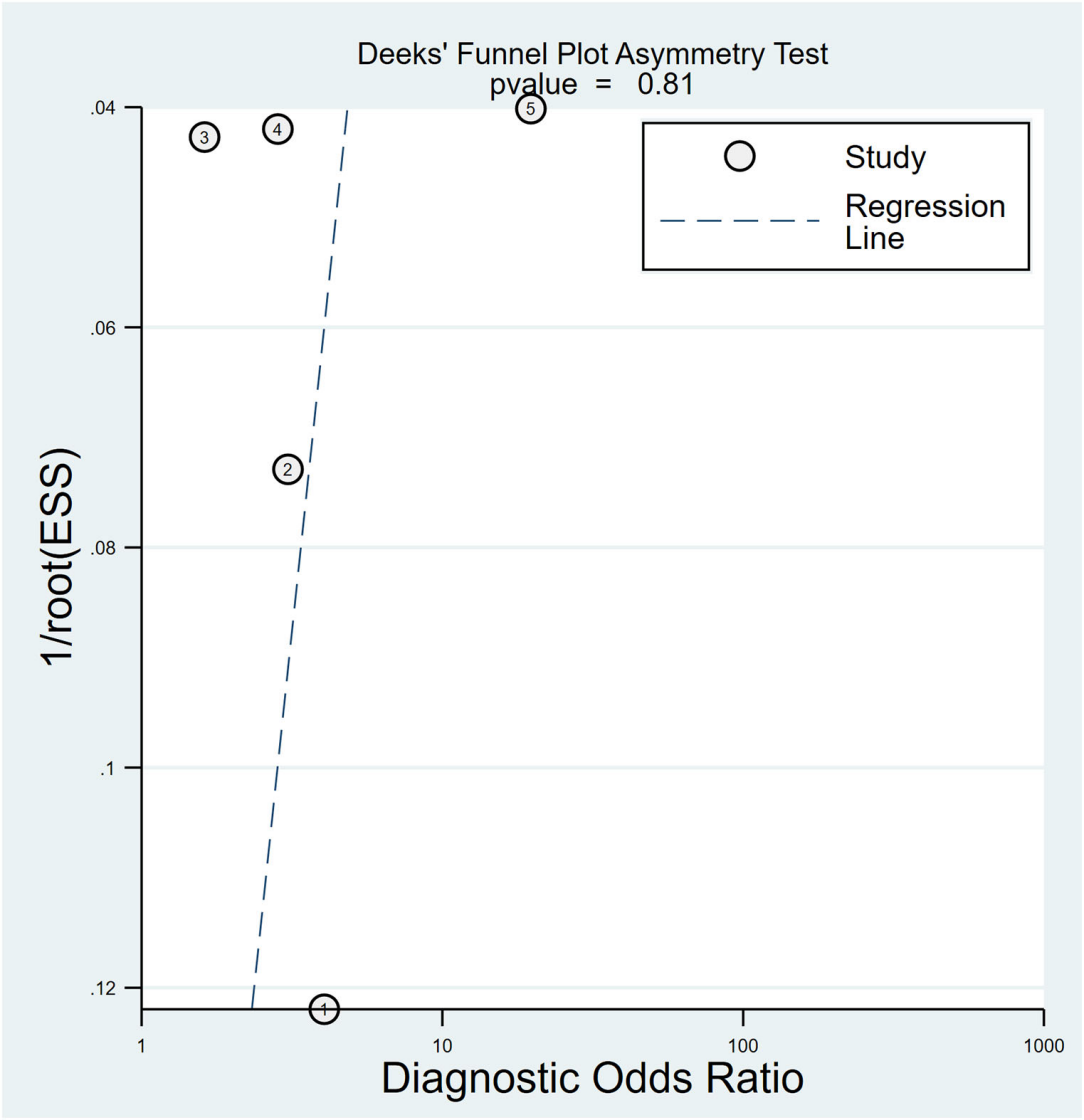
Utilizing Deek’s funnel plot asymmetry test, the probability of publication bias was deemed minimal (p = 0.81).

In relation to the diagnostic effectiveness of the TyG index, the combined sensitivity and specificity values were 0.77 (95% CI 0.59–0.88) and 0.55 (95% CI 0.43–0.68), respectively, accompanied by I^2^ values of 95.1% for sensitivity and 98.29% for specificity (**Figure 5**). The aggregated AUC was 0.69 (95% CI 0.65–0.73) (**Figure 6**). Fagan’s nomogram plot, presented in **Figure 7**, provides a visualization of post-test probabilities derived from the LRs. Specifically, an LR of 2 translates to a marginal increment in the probability of a positive outcome, while an LR of 0.42 signifies a minor reduction in the likelihood of a negative outcome. Notably, Deek’s funnel plot asymmetry test demonstrated a low susceptibility to publication bias (p = 0.81) (**Figure 8**).

## 4 Discussion

On five studies involving 3518 patients with or without DM, patients with a high TyG index were prone to CIN than those with a low index. Regarding the diagnostic efficacy of TyG, the pooled AUC was 0.69, and the pooled sensitivity and specificity were 0.77 and 0.55, respectively. Although several risk factors correlated with CIN development, such as pre-existing kidney disease, diabetes, and heart failure (22–24), whether insulin resistance-associated index could predict CIN following PCI remains unknown. Hereby, this diagnostic meta-analysis highlighted that the TyG index could be a marker that reflects the risk of CIN development in patients receiving PCI.

To date, several risk scores have been established to predict CIN development. For instance, the Mehran contrast nephropathy risk score including hypotension, intra-aortic balloon pump (IABP), heart failure, chronic renal failure, diabetes, old age (>75 years), anemia, and contrast volume, was reported to sensitively predict CIN post-PCI (22). In another study of CIN in patients with ST-segment elevation myocardial infarction who received primary PCI, Koowattanatianchai et al. attempted to set up a simpler predictive model than the Mehran risk score (23). They found that three final predictors, which were ejection fraction of <40%, triple-vessel disease, and use of IABP were the most powerful predictors for the risk stratification of CIN (23). Upon reviewing 16 studies, including 12 prediction models, Silver et al. identified pre-existing chronic kidney disease, age, diabetes, heart failure or impaired ejection fraction, and shock as risk factors for CIN (24). In another meta-analysis, Yin et al. observed that the Maioli score had the best discrimination for CIN occurrence (25, 26). Nevertheless, most studies were based on single-center setting and lacked external validations (25). Thus, a comprehensive review including multiple institutes are necessary to evaluate and discover novel predictive markers for the risk stratification of CIN.

In a study of 1108 patients undergoing PCI for non-ST elevation acute coronary syndrome, the TyG index was identified as an independent predictor of CIN in the multivariate analysis, with a J-shaped association observed between the TyG index and the CIN risk after adjusting for confounders such as DM and kidney function (16). Our pooled meta-analysis also confirmed a significant association between the TyG index and CIN risk (odds ratio: 2.25; I^2^: 35%). However, our meta-analysis, which encompasses five studies with a total of 3518 patients, provides a more substantial evidence base compared to the single study involving 1108 patients (16). This larger pooled dataset enhances the generalizability of the observed association and allows for a more robust analysis of the TyG index’s predictive capacity for CIN. Compared with the TyG index, a recent meta-analysis of four studies involving 1346 patients found that gamma-glutamyl transferase (GGT) levels could potentially predict CIN in patients undergoing cardiac catheterization, showing a significantly higher GGT levels in patients with CIN (odds ratio: 3.21; I^2^:91.93%) (27). In our previous meta-analysis, patients with a low prognostic nutritional index (PNI) demonstrated a higher risk of CIN than those with a high PNI, with an odds ratio of 3.362 (I^2 =^ 89.6%) (28). Although both GGT levels and PNI appear to have a stronger relationship with CIN than the TyG index, the relatively low heterogeneity in the current meta-analysis highlighted that the TyG index may be a reliable predictor of CIN.

Our meta-analysis found that the TyG index had the modest diagnostic accuracy based on the pooled sensitivity of 0.77 and specificity of 0.55. These results may be attributed to the complex, multifactorial mechanisms driving CIN, which are not yet fully elucidated. Proposed pathways for CIN include renal ischemia and hypoxia from altered hemodynamics, direct tubular cytotoxicity, and inflammation/oxidative stress (4, 29, 30). More specifically, contrast agents can reduce renal blood flow through the effects on vasoactive mediators such as nitric oxide and endothelin-1, leading to ischemic injury (4). Contrast media may directly induce tubular epithelial cell damage by increasing intracellular calcium levels, disrupting the mitochondria, and generating reactive oxygen species (29). In addition, the high osmolality and viscosity of some agents can impair tubular cell function and morphology (29). Finally, contrast-mediated CIN may involve free radical formation that damages cell membranes and activation of inflammatory and immunologic cascades (30). Consistently, the observed moderate diagnostic efficacy of the TyG index in predicting CIN might be attributed to the presence of multifactorial mechanisms contributing to CIN. Although the TyG index may not be used in isolation for screening, it may be a supplemental predictor to improve CIN detection.

In current meta-analysis, while studies adjusted for potential confounders such as diabetes and kidney function, other factors like contrast volume and hemodynamic instability were not consistently adjusted for across studies included. Failure to control for all potential mediating and moderating variables may weaken the observed TyG-CIN association. In addition, the heterogeneity between studies regarding patient characteristics, TyG cutoffs, and CIN definitions may have reduced the predictive value of the TyG index. Future studies should focus on optimally adjusting for possible confounders to elucidate the independent contribution of the TyG index to CIN occurrence.

Regarding the pathophysiological link between the TyG index and the risk of CIN, while our study did not directly investigate the underlying mechanisms, we can speculate based on existing literature. Metabolic syndrome, which is characterized by a cluster of conditions including insulin resistance, hypertension, dyslipidemia, and obesity, has been reported in several studies to be a stronger predictor of CIN (31, 32). Given that the TyG index is a recognized surrogate marker for insulin resistance and has been associated with metabolic syndrome (33, 34), it is plausible to consider the TyG index as a potential predictive marker for CIN.

The TyG index has garnered attention because of its potential clinical applications, particularly in the assessment of insulin resistance (9, 12). High TyG index levels are linked to reduced insulin sensitivity (9). This metric can be an initial screening tool for identifying individuals at risk of insulin resistance (9). Furthermore, the TyG index allows for monitoring alterations in insulin resistance and metabolic well-being in response to interventions such as lifestyle modification and medications (9, 12). A low TyG index could indicate enhanced insulin sensitivity and an improved metabolic status (35). The homeostasis model assessment of insulin resistance (HOMA-IR) is another commonly used marker of insulin resistance (36). However, the TyG index has some potential advantages over HOMA-IR. The TyG index requires only fasting glucose and triglyceride levels, which are routinely measured in clinical practice. In contrast, HOMA-IR calculation requires fasting insulin levels, which may not be routinely available. The simpler calculation and widespread availability of the components make the TyG index easily applicable. Furthermore, recent research highlights the TyG index as a superior alternative to HOMA-IR for predicting metabolic syndrome or type 2 diabetes, emphasizing its enhanced efficacy as a surrogate marker of insulin resistance (37, 38).

Several systematic reviews and meta-analyses have examined the relationship between the TyG index and cardiovascular diseases (39–41). A recent meta-analysis of seven studies, contrasting the highest-TyG group with the lowest-TyG group, revealed a notable rise in the risk of heart failure for the higher-TyG group (i.e., HR: 1.21) (39). Another meta-analysis reported that individuals with elevated TyG index levels face an increased risk of cardiovascular disease [i.e., odds ratio: 1.94], more pronounced coronary artery lesions [odds ratio: 3.49], and a less favorable prognosis than those with lower TyG index levels (40). These studies highlight the growing evidence linking the TyG index to increased risks of various cardiovascular diseases. In addition, studies have explored the potential link between the TyG index and nephropathy, particularly in the context of diabetic nephropathy (23, 25, 26). Thus, while the TyG index holds promise across various clinical applications, our meta-analysis suggested that it should not be solely relied upon as a diagnostic tool. Its interpretation should be complemented with other clinical data, including patient history, physical examinations, and supplementary laboratory tests.

Compared with other metabolic predictors, the TyG index had better diagnostic performance for predicting CIN compared with HbA1c, fasting glucose, or triglycerides alone based on the AUC analysis (16). However, the ideal approach for integrating the TyG index into CIN prediction remains unclear, as our meta-analysis relied on aggregate study-level data. A previous study found that adding TyG to the Mehran risk score increased its predictive accuracy from 62.3% to 71.2% and incorporating TyG improved risk reclassification compared with adding fasting blood glucose alone (16). This suggests that the TyG index may have incremental value when combined with established risk factors or prediction models.

This meta-analysis is the first to specifically assess and quantify the TyG index’s overall diagnostic accuracy in predicting the risk of CIN in patients undergoing PCI. While previous research has primarily concentrated on the TyG index’s relationship with diabetic nephropathy, our meta-analysis extends the scope by focusing on its utility in predicting CIN post-PCI. We provide pooled estimates for the sensitivity and specificity of the TyG index, offering a comprehensive view of its diagnostic performance as a predictor for CIN. This meta-analysis not only yields a more accurate assessment of the link between a heightened TyG index and the risk of CIN but also underscores the need for more extensive studies to determine optimal TyG thresholds and to confirm its predictive value across diverse populations.

This meta-analysis has several limitations. First, while showing promise, the evidence base remains limited to just a handful of studies conducted mainly in two countries. Larger scale and more geographically diverse studies are needed to confirm the association between the TyG index and CIN risk. Second, as our meta-analysis found significant heterogeneity between studies, optimal TyG index cutoff values for predicting CIN risk may differ based on patient population characteristics. Careful calibration is necessary before applying proposed cutoffs such as 8.69–9.17 from these studies. Third, this study only investigated the predictive utility of baseline or pre-procedural TyG index. Changes in the TyG index during hospitalization may also correlate with CIN but were not examined here. Finally, the lack of individual patient data precluded more detailed assessment of how the TyG index could enhance existing CIN prediction models. Future pooled analyses should focus on optimally incorporating the TyG index into the risk stratification.

## 5 Conclusion

The results of this meta-analysis showed that patients who received PCI with a higher TyG index were at an increased risk to develop CIN than those with a low index. In addition to the conventional risk factors, the TyG index may be used as an additional predictor of CIN. Nevertheless, CIN can stem from various factors. Therefore, clinical practitioners must take into account all potential risks holistically and evaluate each patient’s situation individually. In addition, the small number of studies identified makes the results preliminary and precludes definitive conclusions. Larger studies are needed to validate the utility of the TyG index and determine appropriate cutoffs.

## Data availability statement

The original contributions presented in the study are included in the article/**Supplementary Material**. Further inquiries can be directed to the corresponding author.

## Author contributions

W-TC: Conceptualization, Writing – original draft, Writing – review & editing. C-CL: Conceptualization, Investigation, Writing – original draft. Y-TH: Data curation, Formal Analysis, Investigation, Software, Writing – original draft. J-YW: Formal Analysis, Methodology, Software, Writing – original draft. W-WT: Investigation, Methodology, Resources, Writing – original draft. K-CH: Writing – original draft, Writing – review & editing. I-WC: Supervision, Writing – original draft, Writing – review & editing. P-HF: Supervision, Writing – original draft, Writing – review & editing.
