# Supplementary material for: Diagnostic efficacy of the triglyceride–glucose index in the prediction of contrast-induced nephropathy following percutaneous coronary intervention
Source: Front Endocrinol (Lausanne). 2023 Nov 22;14:1282675. doi: 10.3389/fendo.2023.1282675 (PMC10703478; doi:10.3389/fendo.2023.1282675)
Supplement: Supplementary file 1 [file Table_1.docx]

**Supplemental table 1.** Search strategy for Databases

|  | Medline | Number of records |
| --- | --- | --- |
| 1 | ("PCI" or "Left Main Disease" or "Coronary Angiograph*" or "coronary artery disease" or "Percutaneous Coronary Intervention" or "Myocardial infarction" or "MI").mp. | 469510 |
| 2 | exp "Percutaneous Coronary Intervention"/ or exp "Coronary Artery Disease"/ or exp "Coronary Angiography"/ | 168265 |
| 3 | ("Triglyceride-Glucose Index" or "Triglyceride-Glucose Indices" or "TyG" or "insulin resistance").mp. | 116780 |
| 4 | ("CI-AKI" or "CA-AKI" or "Contrast-induced acute kidney injury" or "Contrast-Associated Acute Kidney Injury" or "Contrast-Induced Nephropathy" or "Contrast-Associated Nephropathy" or "Acute Renal Insufficiency" or "Acute Kidney Insufficiency" or "Renal Insufficienc*" or "Kidney insufficienc*" or "Acute kidney injury" or "AKI" or "Nephropathy").mp. | 197763 |
| 5 | exp "Acute Kidney Injury"/ or exp "Renal Insufficiency"/ | 203022 |
| 6 | (1 or 2) and 3 and (4 or 5) | 122 |
|  | | |
|  | Embase | Number of records |
| 1 | ("PCI" or "Left Main Disease" or "Coronary Angiograph*" or "coronary artery disease" or "Percutaneous Coronary Intervention" or "Myocardial infarction" or "MI").mp. | 700975 |
| 2 | exp "Percutaneous Coronary Intervention"/ or exp "Coronary Artery Disease"/ or exp "Coronary Angiography"/ | 440075 |
| 3 | ("Triglyceride-Glucose Index" or "Triglyceride-Glucose Indices" or "TyG" or "insulin resistance").mp. | 188344 |
| 4 | ("CI-AKI" or "CA-AKI" or "Contrast-induced acute kidney injury" or "Contrast-Associated Acute Kidney Injury" or "Contrast-Induced Nephropathy" or "Contrast-Associated Nephropathy" or "Acute Renal Insufficiency" or "Acute Kidney Insufficiency" or "Renal Insufficienc*" or "Kidney insufficienc*" or "Acute kidney injury" or "AKI" or "Nephropathy").mp. | 227980 |
| 5 | exp "Acute Kidney Injury"/ or exp "Renal Insufficiency"/ | 492985 |
| 6 | (1 or 2) and 3 and (4 or 5) | 653 |
|  | Cochrane library | Number of records |
| 1 | ("PCI" OR "Left Main Disease" OR "Coronary Angiograph*" OR "coronary artery disease" OR "Percutaneous Coronary Intervention" OR "Myocardial infarction" OR "MI"):ti,ab,kw | 63899 |
| 2 | [mh "Percutaneous Coronary Intervention"] or [mh "Coronary Artery Disease"] or [mh "Coronary Angiography"] | 18291 |
| 3 | ("Triglyceride-Glucose Index" OR "Triglyceride-Glucose Indices" OR "TyG" OR "insulin resistance"):ti,ab,kw | 15682 |
| 4 | ("CI-AKI" OR "CA-AKI" OR "Contrast-induced acute kidney injury" OR "Contrast-Associated Acute Kidney Injury" OR "Contrast-Induced Nephropathy" OR "Contrast-Associated Nephropathy" OR "Acute Renal Insufficiency" OR "Acute Kidney Insufficiency" OR "Renal Insufficienc*" OR "Kidney insufficienc*" OR "Acute kidney injury" OR "AKI" OR "Nephropathy"):ti,ab,kw | 12826 |
| 5 | [mh "Acute Kidney Injury"] OR [mh "Renal Insufficiency"] | 11726 |
| 6 | (#1 OR #2) AND (#3) AND (#4 OR #5) | 0 |
